# Automatic segmentation of the choroid plexuses: Method and validation in controls and patients with multiple sclerosis

**DOI:** 10.1016/j.nicl.2023.103368

**Published:** 2023-03-06

**Authors:** Arya Yazdan-Panah, Marius Schmidt-Mengin, Vito A.G. Ricigliano, Théodore Soulier, Bruno Stankoff, Olivier Colliot

**Affiliations:** a Sorbonne Université, Institut du Cerveau - Paris Brain Institute - ICM, CNRS, Inria, Inserm, AP-HP, Hôpital de la Pitié Salpêtrière, F-75013 Paris, France; b Sorbonne Université, Institut du Cerveau - Paris Brain Institute - ICM, CNRS, Inserm, AP-HP, Hôpital de la Pitié Salpêtrière, F-75013 Paris, France; c Sorbonne Université, Institut du Cerveau - Paris Brain Institute - ICM, CNRS, Inserm, AP-HP, Hôpital Saint-Antoine, F-75012 Paris, France

**Keywords:** Choroid plexus, Segmentation, Deep learning, Multiple sclerosis, Radiologically isolated syndrome

## Abstract

Choroid Plexuses (ChP) are structures located in the ventricles that produce the cerebrospinal fluid (CSF) in the central nervous system. They are also a key component of the blood-CSF barrier. Recent studies have described clinically relevant ChP volumetric changes in several neurological diseases including Alzheimer’s, Parkinson’s disease, and multiple sclerosis (MS). Therefore, a reliable and automated tool for ChP segmentation on images derived from magnetic resonance imaging (MRI) is a crucial need for large studies attempting to elucidate their role in neurological disorders. Here, we propose a novel automatic method for ChP segmentation in large imaging datasets. The approach is based on a 2-step 3D U-Net to keep preprocessing steps to a minimum for ease of use and to lower memory requirements. The models are trained and validated on a first research cohort including people with MS and healthy subjects. A second validation is also performed on a cohort of pre-symptomatic MS patients having acquired MRIs in routine clinical practice. Our method reaches an average Dice coefficient of 0.72 ± 0.01 with the ground truth and a volume correlation of 0.86 on the first cohort while outperforming FreeSurfer and FastSurfer-based ChP segmentations. On the dataset originating from clinical practice, the method reaches a Dice coefficient of 0.67 ± 0.01 (being close to the inter-rater agreement of 0.64 ± 0.02) and a volume correlation of 0.84. These results demonstrate that this is a suitable and robust method for the segmentation of the ChP both on research and clinical datasets.

## Highlights

• The importance of choroid plexuses in neurological diseases has been increasingly recognized.
• Reliable and well-validated methods for choroid plexus segmentation are lacking.
• We propose an automatic tool to segment the choroid plexus with high accuracy.
• The validation included both research and clinical datasets and normal and enlarged choroid plexuses.
• The method opens new perspectives for the study of choroid plexuses on large databases.

## 1 Introduction

Choroid plexuses (ChP) are veil-like structures located in the brain ventricles and composed of a single layer of epithelial cells surrounding a core of capillaries and connective tissue. Their main role is the production of CSF that is renewed several times a day, allowing the maintenance of brain homeostasis through the regulation of fluid and electrolyte balance (Khasawneh et al., 2018). They also contribute to the blood-cerebrospinal fluid barrier, as they integrate signals between the periphery and the central nervous system, and serve as a neuro-immunological interface in physiological and pathological conditions (Ghersi-Egea, 2018, Marques and Sousa, 2015, Schwartz and Baruch, 2014). Finally, they endorse a secretory role, with more than 200 proteins being secreted in the CSF, that may contribute to immunoregulation and neuroprotection (Lun et al., 2015).

These key functions, at the cornerstone of neurodegeneration and neuroinflammation, have pushed forward a research field that aims at unraveling their involvement in neurological diseases such as Alzheimer’s, Parkinson’s disease (Gião et al., 2022, Tadayon et al., 2020), or multiple sclerosis (Ricigliano et al., 2021, Rodríguez-Lorenzo, 2020). On routine MRI sequences such as T1-weighted 3D acquisition, it has been described that ChPs are enlarged in acute and chronic neurological conditions (Althubaity, 2022, Ricigliano et al., 2021, Tadayon et al., 2020), and this enlargement was recently shown to provide a proxy of brain neuroinflammation in preclinical models of multiple sclerosis and patients with multiple sclerosis (Fleischer, 2021, Ricigliano et al., 2021).

To date, the gold standard for ChP segmentation on MRI remains manual annotation, a time-consuming approach that is cumbersome to apply to large cohorts of subjects. Alternative automatic tools are needed and a few solutions for the automatic segmentation of the ChP have been proposed: i) FreeSurfer is the most widely used software for brain segmentation (Fischl, 2012), but the ChP automatic segmentation obtained with FreeSurfer correlated poorly with the ground truth manual segmentation (Ricigliano et al., 2021); ii) A Bayesian Gaussian Mixture Model (GMM)-based approach was shown to outperform FreeSurfer, reaching a Dice coefficient above 0.7 when compared with the ground truth in a healthy control population, but that dropped below 0.6 for diseased subjects (Tadayon et al., 2020); iii) A single 3D U-Net also showed promising performances but the study only included ten subjects (Zhao et al., 2020); iv) finally, an axial multi-layer perceptron yielded very good results both in healthy controls and subjects with multiple sclerosis (Schmidt-Mengin, et al., 2021) but was dependent on the registration of MRIs to standard space and did not outperform a conceptually simpler approach based on the U-Net. Furthermore, with the increase in image resolution, training deep learning models can become a potentially challenging task as hardware limitations (in particular, GPU memory) emerge.

In this study, we aim to develop a simple and highly reliable solution for ChP segmentation, that could be largely applied on T1-weighted MRI sequences acquired in the clinical setting for the diagnosis or follow-up of neurological diseases, and that would require minimal preprocessing steps on images. For this purpose, we have designed a 2-step 3D U-Net that consists of a first ChP segmentation from the whole image, followed by a second segmentation from patches containing ChP. Training and validation were performed in healthy controls and people with multiple sclerosis, with the last validation step achieved on clinical data obtained in subjects with Radiologically Isolated Syndrome (RIS), a preclinical form of MS. This newly developed method will be compared to a standard 3D U-Net (Çiçek et al., 2016) and to the widely used FreeSurfer and FastSurfer (Henschel et al., 2020) software packages.

## 2 Materials and methods

### 2.1 Datasets

For this study, we used two different datasets: a research dataset and a clinical dataset.

The research dataset (further denoted as dataset1) was constituted by gathering data from three prospective studies performed between May 2009 and September 2017 at the Paris Brain Institute (ICM). The dataset1 is composed of 141 participants: 44 healthy controls, 61 patients with relapsing-remitting multiple sclerosis (MS), and 36 patients with progressive MS. Images were acquired on two different Siemens 3 T MRI scanners (Trio and Prisma) with a 32-channel head coil (92 on Trio and 49 on Prisma). The sequence acquired is a 3D T1-weighted magnetization-prepared rapid gradient-echo imaging (MPRAGE) (repetition time = 2300 ms, echo time = 2.98 ms). The studies were approved by the local ethics committees and written informed consent was obtained from all participants (EudraCT no. 2008004174–40 and ClinicalTrials.gov identifiers NCT02305264 and NCT01651520). Further details on the dataset1 can be found in Ricigliano et al. (2021).

The clinical dataset (further denoted as dataset2) was composed of 27 pre-symptomatic MS cases fulfilling the 2009 diagnostic criteria for Radiologically Isolated Syndrome (RIS) (Okuda et al., 2009)followed at the outpatient Neurology clinics of Pitié-Salpêtrière and Saint-Antoine Hospital in Paris, France, between September 2013 and January 2021 and for whom 3D-T1 weighted MRI scans were available. MRI was acquired as part of a clinical routine using non-harmonized protocols on different MRI machines and at different radiological departments (either within or outside the hospitals). Consent for data collection and analysis according to French legislation for non-interventional research was obtained from all subjects (APHP-20210727144630). Further details on dataset2 can be found in Ricigliano et al. (2022).

A summary of the demographics of both datasets can be found in Table 1 and a summary of different machines used for acquisitions can be found in Table 2.

**Table 1:** Demographics. Healthy Controls (HC), presymptomatic multiple sclerosis (RIS), relapsing-remitting multiple sclerosis (RRMS), progressive multiple sclerosis (PMS), Expanded Disability Status Scale (EDSS).

	HC	RIS	All MS	RRMS	PMS
No. of participants	44	27	97	61	36
No. of women	23	17	49	31	18
Age (y)	39 ± 14	42 ± 11	42 ± 12	37 ± 10	50 ± 11
Disease duration (y) (min–max)	N.A	0	4.8 (0.1–23)	4 (0.1–23)	6.5 (0.3–22)
EDSS score	N.A	0	3 (0–7.5)	2 (0–6)	6 (2.5–7.5)

**Table 2:** MRI Machines used for the acquisition of dataset2.

Brand	Model	Count	Total
GE	Optima MR450w (1.5T)	8	16
	SIGNA Explorer (1.5T)	3	
	Signa Artist (1.5T)	3	
	Signa HDxt (1.5T)	1	
	Discovery MR750 (3T)	1	

Siemens	Magnetom Skyra (3T)	5	9
	Magnetom Aera (1.5T)	2	
	Magnetom Avanto Fit (1.5T)	1	
	Magnetom Trio (3T)	1	

Phillips	Ingenia (1.5T)	2	2

### 2.2 Choroid plexus manual segmentation

Before the manual segmentation, all images were corrected for MRI field inhomogeneities using the N4 algorithm implemented in Advanced Normalization Tools (Tustison et al., 2010). On both datasets, the ChP in the two lateral ventricles were segmented by a trained neurologist and corrected by a senior neurologist with long-term expertise in MRI processing (procedure named annotator 1). All segmentations were done using ITK-SNAP (Yushkevich et al., 2006). The portion of choroid plexuses located within the lateral ventricles of the brain was the only one segmented by the two annotators, as done in previous studies (Ricigliano et al., 2022). Indeed, the other parts, located along the roof of the 3rd and 4th ventricles, are hardly and inconsistently visualized. Manual annotation was performed on the axial non-enhanced 3D-T1w images, until the last visible portion within the temporal horn of the lateral ventricles on both sides, then verified and refined on the coronal and sagittal planes. Segmentations were used as ground truth to train the proposed model and evaluate its performance. Finally, for dataset2 only, ChP were segmented a second time by a trained neurologist (annotator 2), independently from the previous annotator. An example of manual segmentation can be found in Fig. 2.

**Fig. 1:**
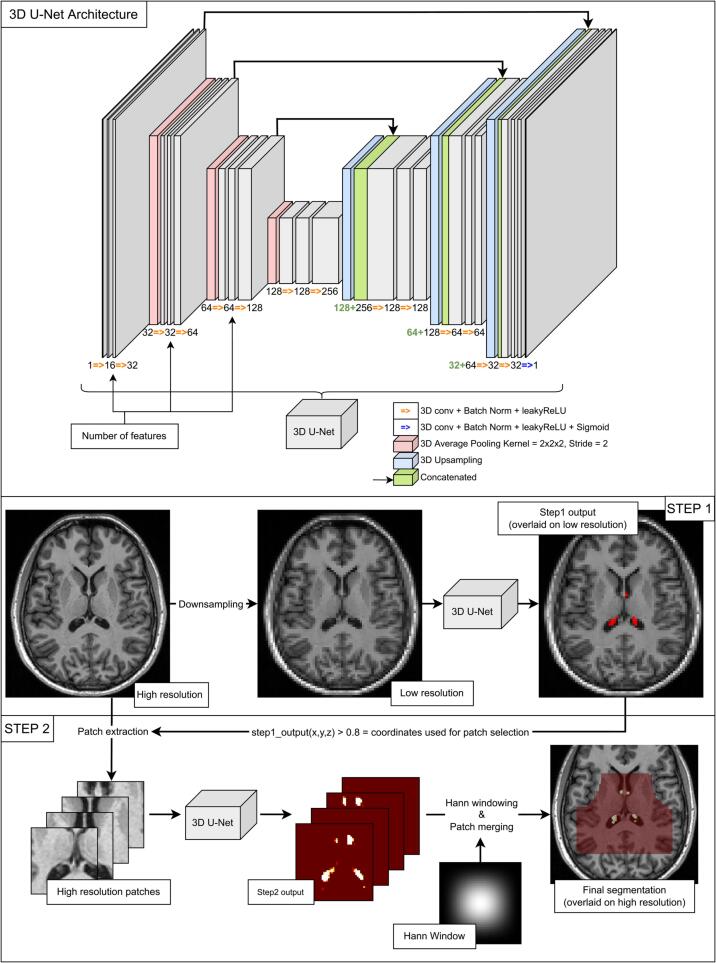
3D U-Net Architecture and 2-step method. Top row: Architecture of the 3D U-Net used in the architecture. Two bottom rows: description of the 2-step method. Middle row: description of step1, «High resolution» corresponds to images with a dimension of 176 × 240 × 256 with voxels of size 1 mm3; «Low resolution» corresponds to images down-sampled to a dimension of 72 × 96 × 104. Step1 output is thresholded at 0.1 for visualization purposes. (2 column fitting image).

**Fig. 2:**
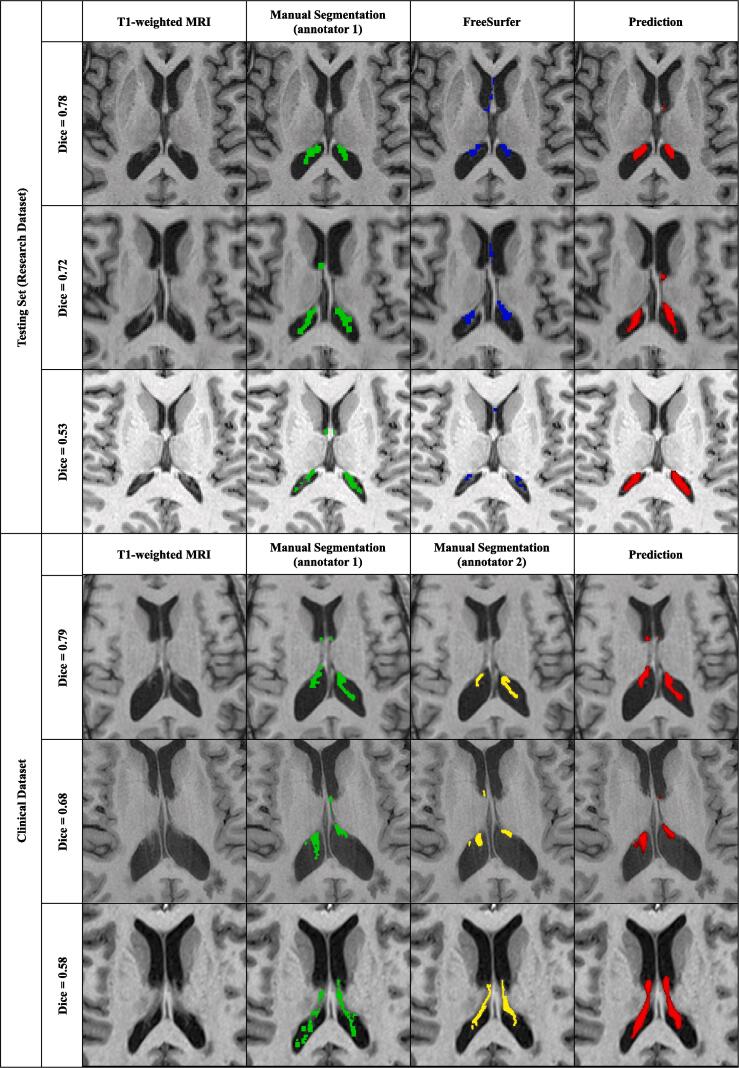
Examples of manual and automatic segmentations on the testing sets from dataset1 and dataset2. Illustrative subjects with the highest, median, and lowest Dice are presented from top to bottom in each dataset box. Each line corresponds to one subject. The manual segmentation of annotators 1 and 2 are respectively in green and yellow. The prediction of the 2-step method trained with data augmentation is presented in red, thresholded at 0.5, and denoted as “prediction”. The reported Dice is between the prediction and the manual segmentation. When there are two annotators, the Dice shown is computed between the prediction and the 1^st^ annotator. (2 column fitting image). (For interpretation of the references to colour in this figure legend, the reader is referred to the web version of this article.). (For interpretation of the references to colour in this figure legend, the reader is referred to the web version of this article.)

### 2.3 Choroid plexus automatic segmentation

#### 2.3.1 Preprocessing

First, images were reoriented to canonical voxel orientation, so that the left–right direction corresponds to the first dimension, the anteroposterior direction to the second dimension, and the infero-superior direction corresponds to the third dimension. Note that we did not perform any registration to a template. Images were then resampled to have an isotropic 1 mm^3^ voxel size and were cropped or padded to match an image dimension of (176, 240, 256), corresponding to the dimensions of the majority of the training dataset (denoted as “HighRes”). Voxel intensities were rescaled to the interval [-1, 1] excluding the lowest and highest 0.5 percentiles.

#### 2.3.2 Proposed method

The method is summarized in Fig. 1. It is composed of two 3D U-Nets (Çiçek et al., 2016, Ronneberger et al., 2015) in cascade, denoted as step1 and step2. This approach will be denoted as “2-step” in the remainder of this manuscript.

Step1 takes as input the whole image down-sampled to a lower size of 72 × 96 × 104 (denoted as “LowRes”). The output probabilities of the first model are used as locations for extracting patches. Patches of size 48 × 48 × 48 are randomly selected around voxels with a probability higher than 80 % to be ChP from the image in its original resolution, with a limit of 500 patches, allowing potential overlap.

Step2 takes said patches as input to segment the ChP. Segmented patches are finally merged to create the final segmentation mask. Patches are merged using a Hann windowing to reduce edge effects (Pielawski et al., 2020). Each patch is therefore multiplied by a Hann window and summed to the output image. In parallel, all Hann windows used to weigh the patches are summed at their corresponding location in an additional mask that will divide the final output to normalize it.

The 3D U-Nets of both steps have the same architecture which is depicted in the first row of Fig. 1. The architecture slightly differs from that of the original 3D U-Net by Çiçek et al. (2016). The convolution blocks use group normalization as it is more reliable for smaller batch sizes (Wu and He, 2018) and a leaky ReLu is used instead of a ReLu to avoid “dead ReLu” effects. The proposed up-convolution was also replaced by an up-sampling, making the model slightly less memory-consuming by reducing the number of trainable parameters. The model has 16 output filters after the first convolution block and 4 levels.

We compared our approach to a standard 3D U-Net applied to the whole image and performed the segmentation in a single step (this approach is denoted as “1-step”). Note that the architecture of this network is the same as that of the previous “2-step” approach. In particular, we made the same modifications to the original 3D U-Net of Çiçek et al. (2016).

The loss function used is the sum of the Sørensen–Dice loss and the binary cross entropy loss (Jadon, 2020) (BCE). Considering two probabilistic or binary segmentations X and Y, the Sørensen–Dice coefficient is defined as:

$$\mathrm{Dice}=\frac{2\sum_{i}{min(x}_{i},y_{i})}{\sum_{i}x_{i}+\sum_{i}y_{i}}withx_{i},y_{i}\in X,Y$$

and the Sørensen–Dice loss as:

DL=1-Dice

Finally, the loss is equal to:

Loss=DL+BCE

The loss of the 2-step approach is then defined as follows:

$${\mathrm{Loss}}_{2-step}=\left\{\begin{array}{cc} {\mathrm{Loss}}_{step1} & \mathrm{if}\;N=0\\ {\mathrm{Loss}}_{step1}+{\mathrm{Loss}}_{step2} & \mathrm{if}\;N>0 \end{array}\right)$$

where N is the number of patch locations found after step1, Lossstep1 is the Loss applied to the LowRes images and Lossstep2 is the Loss applied to the patches. Therefore, in the absence of patch location found following step1, the loss of step2 is ignored and the total loss is equal to the loss of step1. The loss of the 1-step approach is the Loss applied to the HighRes image.

#### 2.3.3 Implementation details

The models were implemented using PyTorch (Paszke, 2019). An Adam optimizer (Kingma and Ba, 2017) was used with an initial learning rate of 1e−3. The learning rate is halved when the validation loss reaches a plateau, defined as a change lower than 1e−3 between two consecutive epochs. Experiments were performed on an Nvidia Tesla V100 32Go graphics card which allows the use of a batch size of 4 LowRes images as input for step1 and a maximum of 16 patches per image extracted from the HighRes images resulting in a batch size of 64 patches as input for step2. The 1-step approach taking as input HighRes images is trained with a batch size of 1, which is the maximum that could fit into memory.

#### 2.3.4 Optional data augmentation

Augmentations were optionally applied. The augmentations were computed using TorchIO (Pérez-García et al., 2021) and are summarized in Table 3. Elastic deformations were not used as an augmentation method due to high computation times and limited availability of computing resources. This data augmentation procedure is applied to simulate artifacts and types of noises encountered when routinely working with MRI and virtually augment the size of our training set.

**Table 3:** Data augmentations applied.

Augmentation	Probability	Characteristics
Left-Right flip	0.5	–
Affine transformations	0.3	max scaling = 0.3; max rotation = 15°
Image anisotropy	0.3	can be applied along all axes; max down sampling factor = 2
MRI motion artifact (Shaw et al., 2019)	0.3	max rotation = 15°; max translation = 15 mm; max number of movements = 2
MRI ghosting artifacts	0.3	number of ghosts = 2; can be along all axis
MRI spike artifacts	0.3	number of spikes = 1;
MRI bias field	0.3	maximum magnitude = 0.5; polynomial order = 3
Gaussian Noise	0.3	mean = 0; standard deviation = 1
Contrast modification	0.3	log(gamma) = 0.3

### 2.4 Experiments

#### 2.4.1 Training and validation procedures

The dataset1 was split into 72 subjects in the training set, 19 subjects in the validation set, and 50 subjects in the testing set. The testing set (from dataset1) was left untouched until the end and was only used to evaluate the performances. For both architectures, training was done with and without data augmentation. The training was performed during 200 epochs for the 2-step method, and 100 epochs for the 1-step method. The dataset2 was not used for training and was only used as a second test set.

The python package Weights&Biases (Biewald, 2020) was used for experiment tracking, visualization, and memory monitoring.

#### 2.4.2 Performance evaluation

To evaluate the performance of an architecture, the following scores have been used:

Dice $=\frac{2\sum_{i}{min(x}_{i},y_{i})}{\sum_{i}x_{i}+\sum_{i}y_{i}}$; Recall $R=\frac{\sum_{i}x_{i}y_{i}}{\sum_{i}y_{i}}$; Precision $P=\frac{\sum_{i}x_{i}y_{i}}{\sum_{i}x_{i}}$;

Volume error rate $\mathrm{VER}=\frac{\sum_{i}x_{i}-y_{i}}{\sum_{i}y_{i}}$Absolute volume error rate $\mathrm{AVER}=\left|VER\right|$where $x_{i},y_{i}\in X,Y$ are respectively the prediction and the ground truth.

The recall, precision, volume error rate, and absolute VER, are common metrics extracted to evaluate performances in segmentation tasks. Just like the Dice coefficient, they are a representation of the similarity of our predicted mask with the ground truth. The recall nuances this result by showing the percentage of true positives over the total number of positive voxels found. The higher the recall, the less likely we are to miss positive voxels and the more sensible the model is. Similarly, the precision nuances the information given by the Dice coefficient by evaluating the percentage of the predicted mask that is correct. The higher the precision, the less the model is likely to select voxels wrongly. Finally, VER and AVER, as we are looking for a reliable method for choroid plexus volume estimation, are all the more relevant and informative for the future task this method might be applied for.

Results were reported as mean ± standard error of the mean (SEM). To assess the influence of each experiment on the outcome Dice coefficient, a linear mixed effect model was fitted using the lme4 package (v1.1–31) in R version 4.2.2. For each linear mixed model, we tested the effect of data augmentation, model architecture, and their interaction. We, therefore, used a Bonferroni significance threshold of 0.017 correcting for 3 tests. To assess statistical differences between the scores, paired Student T-tests with a Bonferroni correction for multiple comparisons were used. Note that statistical testing was only performed on the testing datasets. Finally, correlations between predicted volumes and manual segmentations are reported using Pearson’s r.

#### 2.4.3 Comparison with other approaches

We compared the results obtained using our approach to those obtained using: 1) the aforementioned 1-step approach; 2) FreeSurfer (Fischl, 2012) version 6.0.0; 3) FastSurfer (Henschel et al., 2020) version 1.0.0.

## 3 Results

### 3.1 Memory usage, training time, and inference time

The Nvidia Tesla V100 GPUs used have 32 GB of available memory. During the training of the two architectures, the 1-step method reached 93.23 % of available memory with a batch size of 1 while the 2-step method reached 94.95 % of available memory with a batch size of 4 for step1 and a batch size of 64 for step2.

Without data augmentation, the 2-step method averaged at 1.57 ± 0.03 min/epoch (mn/e) versus 3.39 ± 0.01 mn/e. With data augmentation, training times were increased to 4.56 ± 0.04 mn/e for the 2-step and 5.18 ± 0.01 mn/e for the 1-step method. Inferring on images takes approximately 30 s with the 1-step and 15 s with the 2-step on CPU and less than 10 s on GPU for both methods. All times include opening images, preprocessing, inferring, postprocessing, and saving inferred masks.

Tests were also performed with float 16 precision, but this led to underflow errors and the apparition of outlying numbers.

### 3.2 Segmentation performances

Fig. 2 displays examples of manual and automatic segmentations for different subjects of the testing sets from dataset1 and dataset2, corresponding respectively to low, average, and high Dice coefficients.

Results on the validation set of dataset1 are reported in Table 4 and results on the testing set of dataset1 in Table 5. For all architectures, performances are comparable between the validation and testing sets, indicating that the validation set was not overfitted. The average Dice is around 0.7 for all deep learning methods. For all metrics, the proposed deep learning methods provided considerably better performances compared to FreeSurfer and FastSurfer.

**Table 4:** Results on the validation set from dataset 1. Results are presented as mean ± standard error of the mean across the dataset. Volume Error Rate denoted as VER, absolute VER denoted as AVER. Best performances are denoted in bold face.

Method	Data Augmentation	Dice	Recall	Precision	VER	AVER	Pearson r
1-step	no	**0.73 ± 0.01**	**0.73 ± 0.02**	0.75 ± 0.02	**0.04 ± 0.05**	**0.16 ± 0.03**	0.76
	yes	0.71 ± 0.01	0.70 ± 0.02	0.74 ± 0.02	0.08 ± 0.04	**0.16 ± 0.03**	0.82

2-step	no	**0.73 ± 0.01**	0.70 ± 0.02	**0.76 ± 0.02**	0.11 ± 0.05	0.18 ± 0.04	0.73
	yes	0.70 ± 0.01	0.68 ± 0.02	0.74 ± 0.01	0.12 ± 0.04	**0.16 ± 0.03**	**0.86**

**Table 5:** Results on the testing set from dataset1. Results are presented as mean ± standard error of the mean across the dataset. Data augmentation does not apply to FreeSurfer and FastSurfer. Volume Error Rate denoted as VER, absolute VER denoted as AVER. Best performances are denoted in boldface.

Method	Data Augmentation	Dice	Recall	Precision	VER	AVER	Pearson r
1-step	no	**0.73 ± 0.01**	**0.72 ± 0.01**	0.75 ± 0.01	**0.07 ± 0.03**	**0.18 ± 0.02**	0.84
	yes	0.71 ± 0.01	0.68 ± 0.02	**0.76 ± 0.01**	0.17 ± 0.04	0.25 ± 0.04	0.77
2-step	no	0.72 ± 0.01	0.70 ± 0.01	**0.76 ± 0.01**	0.12 ± 0.03	0.19 ± 0.02	**0.86**
	yes	0.69 ± 0.01	0.65 ± 0.01	**0.76 ± 0.01**	0.20 ± 0.04	0.26 ± 0.04	0.78
FreeSurfer		0.33 ± 0.01	0.42 ± 0.01	0.29 ± 0.01	−0.29 ± 0.03	0.32 ± 0.02	0.65
FastSurfer		0.35 ± 0.01	0.40 ± 0.01	0.32 ± 0.01	−0.18 ± 0.03	0.25 ± 0.02	0.59

Results on dataset2 are reported in Table 6. Performances were in general lower than those obtained on dataset1. On dataset2, average Dice coefficients between all tested methods and annotator1 ranged from 0.61 to 0.67 and from 0.56 to 0.59 between all tested methods and annotator 2. On dataset1 however, Dice coefficients between predictions and manual segmentations ranged from 0.69 to 0.73. The 1-step and 2-step methods also tended to provide higher segmentation metrics on the dataset2 segmented by the first annotator compared to the second annotator (cf. Table 7, supplementary materials). These differences are significant for methods trained with data augmentation (p = 4.23E−03 for 1-step, p = 4.88E−04 for 2-step; corrected statistical threshold at 1.25E−02). Finally, the automatic segmentation performance was of the same order of magnitude as the inter-rater variability (mean Dice = 0.64 ± 0.02 SEM for inter-rater agreement).

**Table 6:** Results on dataset2 (testing clinical dataset). Results are presented as mean ± standard error of the mean across the dataset. Volume Error Rate denoted as VER, absolute VER denoted as AVER. Best performances respective to each annotator are denoted in boldface.

Method	Data Augmentation	Annotator	Dice	Recall	Precision	VER	AVER	Pearson r
1-step	no	1	0.61 ± 0.02	0.63 ± 0.02	0.63 ± 0.03	**0.01 ± 0.06**	0.24 ± 0.03	0.68
		2	0.56 ± 0.02	0.49 ± 0.02	0.69 ± 0.03	0.49 ± 0.10	0.60 ± 0.08	0.47
	yes	1	0.64 ± 0.01	0.58 ± 0.02	0.74 ± 0.02	0.32 ± 0.06	0.34 ± 0.05	0.64
		2	0.56 ± 0.02	0.44 ± 0.02	0.80 ± 0.01	0.94 ± 0.11	0.94 ± 0.11	0.48

2-step	no	1	0.62 ± 0.02	**0.64 ± 0.02**	0.61 ± 0.03	−0.04 ± 0.05	**0.19 ± 0.03**	0.75
		2	0.56 ± 0.02	**0.51 ± 0.02**	0.68 ± 0.03	**0.42 ± 0.09**	**0.51 ± 0.07**	0.52
	yes	1	**0.67 ± 0.01**	0.62 ± 0.01	**0.75 ± 0.02**	0.22 ± 0.03	0.24 ± 0.03	**0.84**
		2	**0.59 ± 0.02**	0.47 ± 0.02	**0.81 ± 0.01**	0.80 ± 0.08	0.80 ± 0.08	**0.62**

Inter-rater agreement			0.64 ± 0.02	0.78 ± 0.01	0.55 ± 0.02	−0.29 ± 0.03	0.29 ± 0.03	0.64

**Fig. 3:**
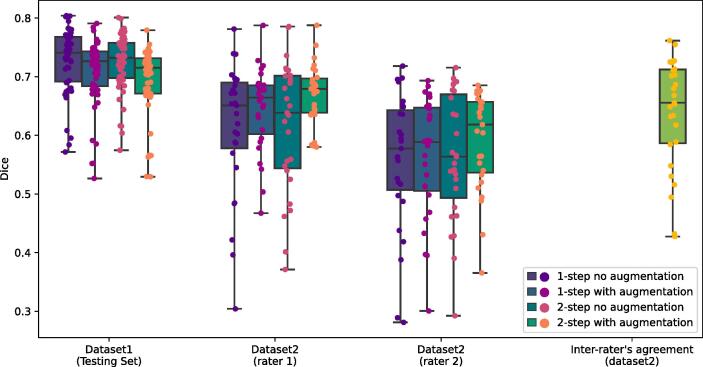
Results on the independent testing set of dataset1 and dataset2. Lower and upper whiskers represent the minimum and maximum values observed in the dataset. Boxes are bound by the first quartile at the bottom and the third quartile at the top with the center line representing the median of the distribution. (2 column fitting image).

A comparison between the different deep learning approaches (1-step, 2-step, with and without data augmentation) is presented in Fig. 3. Data augmentation lowers the Dice coefficient by 0.02 on dataset1 (p = 2.24e−12) as estimated from the linear mixed-effect model. No other tests indicated a significant effect of the method, the data augmentation, or their interaction.

## 4 Discussion

This study presents a simple and reliable method for the automatic segmentation of ChPs called “2-step 3D U-Net”. The 2-step 3D U-Net provides excellent segmentation results that reach performances comparable to a regular 3D U-Net while being less memory hoarding. It advantageously requires minimal preprocessing, without any registration procedure. The 2-step 3D U-Net was demonstrated to be effective on heterogeneous clinical data, including healthy and non-healthy subjects, opening the perspective of broad applications aiming at measuring ChP volume in clinical and research datasets and for routine patient monitoring.

ChP volumetric changes have indeed been associated with a variety of neurological disorders such as depression (Althubaity, 2022), stroke (Egorova et al., 2019), Alzheimer’s disease (Choi et al., 2022), Parkinson’s disease (Tadayon et al., 2020), and multiple sclerosis (Müller, 2022, Ricigliano et al., 2021). Recently, ChP enlargement was even found to discriminate multiple sclerosis from neuromyelitis optica spectrum disorder (Müller, 2022) and proposed as an early imaging biomarker in preclinical forms of multiple sclerosis (Ricigliano et al., 2022). There is therefore an increasing need for reliable segmentation methods of the ChPs to foster research in large datasets and further explore whether ChP volumetry could become a potential diagnostic or prognostic biomarker.

To date, only a few automatic segmentation methods of the ChP have been proposed. Tadayon et al. (2020) have introduced an automatic procedure based on Bayesian Gaussian Mixture Models (GMM) that was subsequently tested on multiple datasets. Their pipeline included 4 steps: segmentation of the lateral ventricles through FreeSurfer; application of a Bayesian GMM to separate 2 classes; smoothing; and finally, a second Bayesian GMM aimed at discriminating the ChP from the background CSF through the selection of the class with the highest mean intensity value. This approach mostly relies on voxel intensity, and no spatial information was considered apart from the initial selection of voxels inside the lateral ventricles. Such an approach could therefore be susceptible to artifacts changing voxel intensities at high spatial frequencies (such as motion, ghosting, and spiking). The GMM outperformed FreeSurfer and provided a Dice coefficient that exceeded 0.7 when segmenting ChPs on non-enhanced MRIs from the Human Connectome Project (Van Essen et al., 2013). However, results dropped below 0.6 on subjects coming from the ADNI cohort (Petersen et al., 2010). Furthermore, it was not tested on clinical routine data.

The axial multi-layer perceptron, recently developed by Schmidt-Mengin et al, yielded performance comparable to a 3D U-Net, particularly the 2-step U-Net presented in our study. However, this approach required a preprocessing of the image through Clinca’s “t1-linear” (Routier et al., 2021)which consists of a correction of MRI bias field through the N4ITK algorithm (Tustison et al., 2010) and registration to standard space, and its dissemination for routine use would imply time-consuming processing steps. Moreover, it is conceptually more complex than a U-Net while providing higher performances.

Finally, Zhao et al. (2020) applied an optimized 3D U-Net. Their method was trained using only 10 MRIs from healthy females. The algorithm was not tested on patients, known to present volumetric changes of the ChP. Their method reached a Dice coefficient of 0.732 ± 0.046 (mean ± standard error of the mean) in the healthy control population. No further testing of their algorithm was performed on other datasets, containing pathological subjects.

Our 2-step 3D U-Net provided similar segmentation performances compared to a regular 3D U-Net, yielding a Dice coefficient of 0.70 vs 0.73. However, the non-significance of p-values does not imply the absence of differences between the methods. A larger test set would provide more statistical power to detect those potential differences. Nevertheless, the overall segmentation performances are close, with the best method alternating between the 1-step and the 2-step according to the task at hand, and the standard errors of the mean remain small, allowing us to believe that the overall performances of the models are comparable. It outperforms the segmentation provided by FreeSurfer by a vast margin. The poor performance of FreeSurfer could partly be due to the fact that their template was built using different guidelines. Indeed, it is difficult to define the ChP boundaries unambiguously, as illustrated by the inter-rater variability. However, visual inspections of FreeSurfer results revealed that there are clear areas of false positives. Thus, it is unlikely that its poor performance is only due to the template. Also, we did not consider SAMSEG (Puonti et al., 2016) which could be a way to improve the FreeSurfer-based ChP segmentation. This is left for future work. In addition, the models have been trained using both healthy subjects and MS patients and have been exposed to a wide range of ChP volumes. Interestingly, data augmentation reduced the loss of performance on dataset2 compared to the models trained without it (Dice = 0.67 with data augmentation vs 0.59 without data augmentation for the 2-step and 0.64 vs 0.56 respectively for the 1-step) and the 2-step method trained with data augmentation provided the best volumetric correlations on dataset2 (0.84 with annotator 1 and 0.62 with annotator 2), emphasizing its optimal reliability for further volumetric analysis of ChPs in large cohorts of subjects. Of note, the first step applied in our protocol was the detection of ChP-containing patches, thus overcoming potential bias linked to class imbalance. By considering spatial information in the image, preprocessing steps such as ventricle segmentation through FreeSurfer or registration to a standard space could be avoided. Another advantage of the 2-step method was the reduced memory consumption required during training, which allowed the use of larger batch sizes compared to the 1-step (4 vs 1). Taken together, the 2-step 3D U-Net fulfills many prerequisites for large clinical applications.

Our study has the following limitations. We acknowledge that the trained models learned slightly better segmentation patterns from annotator 1, which were available for both datasets, than the one from annotator 2, available only for dataset2, which may suggest a slightly reduced reproducibility of the method when heterogeneous real-life data are considered. Also, automatic volumetric analysis of ChPs may miss the presence of microcysts or calcifications, introducing a bias for a minority of subjects. Very few subjects in our datasets presented calcifications or cysts in the ChP so the performance of the methods in those specific cases could not be assessed. Training on a higher number of subjects well characterized for microcysts or calcifications will further benefit the model’s generalizability. Moreover, while the sequence used for segmentation, 3D-T1w MPRAGE MRI, is widely available in all imaging research protocols, it is, however, not the ideal sequence to visualize ChP. First T1w images enhanced with gadolinium allows following the ChP along the walls of the hippocampus. As contrast-enhanced T1w acquisition was not available for all MS subjects and was never performed in healthy subjects, we could neither assess the methods' performance on this modality nor train the model to segment the ChP. Similarly, other T1w imaging modalities such as the spin-echo could not be studied due to unavailability in the dataset. Then, on fluid-attenuated inversion recovery T2-weighted (FLAIR) MRI, thanks to the fluid attenuation, ChP contrast with surrounding CSF is highly enhanced. However, our datasets did not consistently contain 3D FLAIR images. Nevertheless, 3D FLAIR is nearly becoming ubiquitous in imaging protocols and ChP segmentation using this sequence could be of great interest. Finally, no test–retest reproducibility assessment of the method was possible as no short-term rescanning of the subjects was available.

In conclusion, our 2-step method allows excellent segmentation performances both on research and clinical datasets. Such a method could therefore be applied to a further study exploring the role of ChPs in various neurological diseases. Being an automated segmentation method, this protocol could be used to analyze a large amount of data, in cohorts where manual segmentation would be too time-consuming. This fast and easy-to-use tool would allow the extraction of a simple imaging biomarker with potential interest in many neurological diseases.

## Declaration of Competing Interest

The authors declare that they have no known competing financial interests or personal relationships that could have appeared to influence the work reported in this paper.

## Data Availability

Codes will be made available upon request.
