# Supplementary material for: Automatic segmentation of the choroid plexuses: Method and validation in controls and patients with multiple sclerosis
Source: Neuroimage Clin. 2023 Mar 6;38:103368. doi: 10.1016/j.nicl.2023.103368 (PMC10011049; doi:10.1016/j.nicl.2023.103368)
Supplement: Supplementary Data 1 [file mmc1.docx]

# Supplementary materials

| Dataset | Method 1 | Method 2 | Dice 1 | Dice 2 | T-stat | P-val |
| --- | --- | --- | --- | --- | --- | --- |
| Testing set (dataset1) | 1-step no augmentation | 1-step with augmentation | 0.73±0.01 | 0.71±0.01 | 1.85 | 6.80E-02 |
|  | 1-step no augmentation | 2-step no augmentation | 0.73±0.01 | 0.72±0.01 | 0.38 | 7.04E-01 |
|  | 1-step no augmentation | 2-step with augmentation | 0.73±0.01 | 0.69±0.01 | 2.94 | 4.15E-03 |
|  | 1-step with augmentation | 2-step no augmentation | 0.71±0.01 | 0.72±0.01 | -1.56 | 1.22E-01 |
|  | 1-step with augmentation | 2-step with augmentation | 0.71±0.01 | 0.69±0.01 | 1.05 | 2.96E-01 |
|  | 2-step no augmentation | 2-step with augmentation | 0.72±0.01 | 0.69±0.01 | 2.70 | 8.15E-03 |
| Testing set (dataset2, rater1) | 1-step no augmentation | 1-step with augmentation | 0.61±0.02 | 0.64±0.01 | -1.23 | 2.24E-01 |
|  | 1-step no augmentation | 2-step no augmentation | 0.61±0.02 | 0.62±0.02 | -0.10 | 9.21E-01 |
|  | 1-step no augmentation | 2-step with augmentation | 0.61±0.02 | 0.67±0.01 | -2.37 | 2.18E-02 |
|  | 1-step with augmentation | 2-step no augmentation | 0.64±0.01 | 0.62±0.02 | 1.12 | 2.69E-01 |
|  | 1-step with augmentation | 2-step with augmentation | 0.64±0.01 | 0.67±0.01 | -1.46 | 1.51E-01 |
|  | 2-step no augmentation | 2-step with augmentation | 0.62±0.02 | 0.67±0.01 | -2.25 | 2.90E-02 |
| Testing set (dataset2, rater2) | 1-step no augmentation | 1-step with augmentation | 0.56±0.02 | 0.56±0.02 | -0.14 | 8.89E-01 |
|  | 1-step no augmentation | 2-step no augmentation | 0.56±0.02 | 0.56±0.02 | -0.19 | 8.46E-01 |
|  | 1-step no augmentation | 2-step with augmentation | 0.56±0.02 | 0.59±0.02 | -1.23 | 2.25E-01 |
|  | 1-step with augmentation | 2-step no augmentation | 0.56±0.02 | 0.56±0.02 | -0.06 | 9.50E-01 |
|  | 1-step with augmentation | 2-step with augmentation | 0.56±0.02 | 0.59±0.02 | -1.17 | 2.48E-01 |
|  | 2-step no augmentation | 2-step with augmentation | 0.56±0.02 | 0.59±0.02 | -1.05 | 2.97E-01 |

Table 7. Statistical differences of the segmentation methods in the research and clinical datasets. Dices are presented as mean ± standard error of the mean across the dataset. “DiceN” corresponds to the performance of “Method N”. The test performed is a paired student’s T-test. The P-value threshold for significance after Bonferroni correction is 2.8E-03. There was no statistically significant difference after correction.

| Method | Data  Augmentation | Dice rater1 | Dice rater 2 | T-stat | P-val |
| --- | --- | --- | --- | --- | --- |
| 1-step | no | 0.61±0.02 | 0.56±0.02 | 1.79 | 3.15E-01 |
|  | yes | 0.64±0.01 | 0.56±0.02 | 3.47 | **4.23E-03** |
| 2-step | no | 0.62±0.02 | 0.56±0.02 | 1.76 | 3.39E-01 |
|  | yes | 0.67±0.01 | 0.59±0.02 | 4.15 | **4.88E-04** |

Table 8. Statistical differences of the segmentation performances on the clinical dataset as a function of the rater. Dices are presented as mean ± standard error of the mean across the dataset. “Dice rater N” corresponds to the performance of the method’s prediction when compared to the manual segmentation of the annotator N. The test performed is a paired student’s T-test. The P-value threshold for significance after Bonferroni correction is 1.25E-02. Statistically significant differences are marked in bold.
